# Molecular life sciences in the era of the Fourth Industrial Revolution: sequencing, multi-omics and artificial intelligence

**DOI:** 10.1042/ETLS20253019

**Published:** 2025-12-12

**Authors:** Lee Keenan, R. Lorna Younger, Paul R. Race, Matt Bawn

**Affiliations:** 1 School of Natural and Environmental Sciences, Newcastle University, Newcastle upon Tyne, NE1 7RU, U.K

**Keywords:** biotechnology, computational biochemistry, DNA sequencing, genomics, transcriptomics

## Abstract

The onset of the Fourth Industrial Revolution has catalysed a fundamental shift in how research within the molecular life sciences is approached and undertaken. Over the past decade, a multitude of nascent enabling technologies have progressed to maturity and have become irreversibly embedded in laboratory practice. Artificial intelligence (AI) has become a mainstay within the molecular sciences, facilitating major advances across a multitude of sub-disciplines, including synthetic biology, industrial biotechnology and drug discovery. One area where this impact is being particularly felt is within multi-omics, where the marriage of AI with low-cost high-throughput sequencing is delivering unprecedented advances, allowing large and often complex datasets to be deconvoluted on timescales previously considered unimaginable. In this mini-review, we outline how the integration of AI into multi-omics has been realised and forecast future trajectories for research in this important area.

## Introduction

Research in the molecular life sciences continues to benefit from significant and continual technological advances. As technology develops, there is a continuous trend of compounding progress, with each advance serving to reduce the period of time until the next. This principle, first described by Alan Turing [1], is now widely recognised as the law of accelerating returns and describes the exponential increase in technological advancement over time.

**Figure 1:**
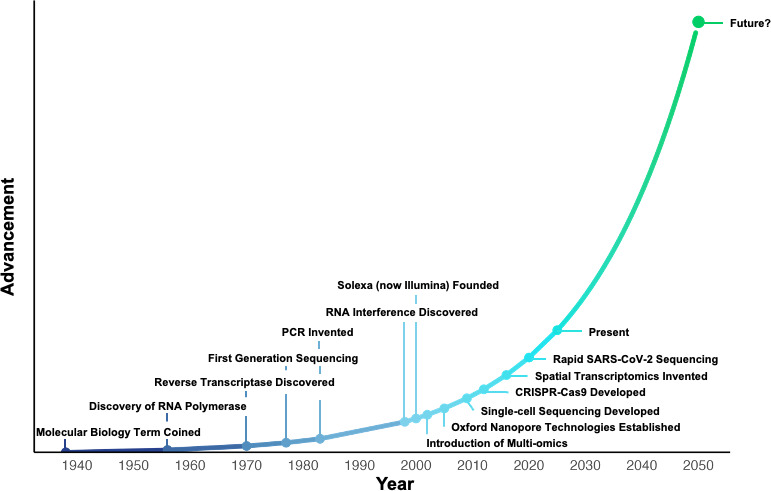
The proposed law of accelerating returns as applied to the molecular life sciences. Each technological innovation shortens the period before the next.

Turing’s principle has proven to be directly applicable in the molecular life sciences (Figure 1), where the adoption of artificial intelligence (AI) methods is anticipated to drive a trajectory of exponential growth. Improvements in AI technology are being increasingly recognised as an indicator of the Fourth Industrial Revolution (4IR) [2]. In contrast with the preceding Third Industrial Revolution, which saw the transition of the internet from specialised networks into a ubiquitous tool for information sharing, communication and the concomitant availability of large amounts of data to the general population [3,4], 4IR is founded on data identification, literacy and analysis, at both a speed and scale previously considered unimaginable. In addition, 4IR embeds the FAIR (findable, accessible, interoperable and reusable) data principles, which seek to safeguard data availability and usability within research practice [5].

One area where AI is having a major impact within the life sciences is in the analysis of sequencing data. The past 50 years have seen substantial advances in sequencing technologies, with a transition from short-read low-throughput methods, characterised by technically challenging and labour-intensive data analysis pipelines, to the current ‘sequence-by-synthesis’ approach. AI-based analysis methods are emerging as integral components of modern sequencing workflows, reducing the increasing burden of large biological datasets generated by state-of-the-art sequencing platforms. This new age of sequencing has revolutionised clinical diagnostic approaches, positively impacting patient outcomes by applying AI, machine learning (ML) and deep learning (DL) models for accurate spatiotemporal analysis of disease pathogenesis. In response to AI’s continuing impact, scientific organisations are beginning to adapt to the reality of operating in the era of 4IR. Consequently, ambitious predictions have been made as to what the future of the molecular life sciences may look like, with evidence pointing towards a future in which AI and automation play an increasingly prominent role in research and innovation.

Despite widespread enthusiasm for the adoption of AI within sequencing and multi-omics applications, coherent concerns have been raised regarding the wider technical and ethical implications of this approach. Unease relating to the curation, accessibility and commercial exploitation of clinical datasets emerging from AI analytics persists, along with disquiet regarding the impact of AI-enabled processes on the global life sciences workforce. To address these issues and to provide a historical context for AI use in the contemporary molecular life sciences, here we outline the transformative contributions that sequencing, multi-omics and AI are making to the 4IR. We propose a requirement for the effective integration of these methods into research and clinical practice, and address the validity of concerns relating to the widespread adoption of these nascent technologies.

### Integration of AI into molecular life science research

AI is the ability of a computer or machine to complete actions which usually require human intelligence. This can range from simple tasks such as making a phone call to complex activities involving the analysis of large datasets or multifactorial decision making. The growing presence of AI has been described as an indicator of 4IR [2,6–8]. The Third Industrial Revolution, often termed ‘the information age’ or ‘the digital age’, was largely characterised by a rise in both access and usage of the internet and the World Wide Web [3,4]. When the internet was popularised, it became possible to access a superabundance of information, including scientific data, almost instantaneously, and with minimal effort. As the quantity of published data continues to rise [9], it will inevitably become more difficult to extract the most relevant information pertaining to a specific topic. However, modern AI-driven models make it possible for this to be achieved within modest timescales.

The underlying algorithms driving AI models are designed to identify relevant information amongst a superfluity of ‘noise’, affording an optimised method for automated data searching [10]. This AI-assisted method of information synthesis expedites data discovery and makes findings more rapidly actionable. By integrating AI-driven processes into research and clinical workflows, data-driven decision making, hypothesis generation and patient diagnosis can be undertaken rapidly and in response to urgent need [11]. However, despite significant progress, challenges remain, including a lack of standardised processes and concerns relating to ethical and legal supervision [12]. Public distrust in AI serves to fuel an undoubted need for the adoption of explainable AI, which informs users of the reasoning employed in AI decision making. High-stakes scenarios which involve AI decision making should be structured to identify potential biases and properly assign accountability.

The use of AI has proven particularly impactful in drug discovery, where up to 90% of drugs in phase I, II or III trials fail to transition to clinical use [13]. A significant hurdle within drug discovery from natural sources is the rediscovery of known compounds [14]. As a result, it is important to employ dereplication steps during the discovery process to rapidly identify known compounds such that they can be deprioritised for further study. AI systems can judiciously match mass spectrometry or nuclear magnetic resonance profiles against existing reference libraries using advanced signal processing to improve signal-to-noise ratios and correct overlapping signals [15]. This approach significantly accelerates the rate of compound dereplication and, in doing so, mitigates wasted research effort.

DL is a sophisticated form of ML that uses artificial neural networks to identify patterns in large data sets, allowing human decision-making processes to be performed autonomously [16]. DL-driven pattern recognition methods have been employed within drug discovery programmes to prioritise individual compounds or groups of compounds for clinical development, with this approach reported to outperform medicinal chemists by up to 54-fold [17].

Advanced pattern recognition, including DL-based methods, can identify regions of genomic data indicative of biosynthetic gene clusters. This information can be used to directly inform natural product drug discovery efforts, which can be further expedited via integration with an AI-guided dereplication approach. These methodical AI algorithms can merge, interpret, analyse and produce data at rates much faster and more accurately than is achievable using non-AI-based methods. In an age of ‘Big Data’, AI has become an indispensable tool of the contemporary bioscientist.

### Sequencing technologies – the great enabler of 4IR

**Figure 2:**
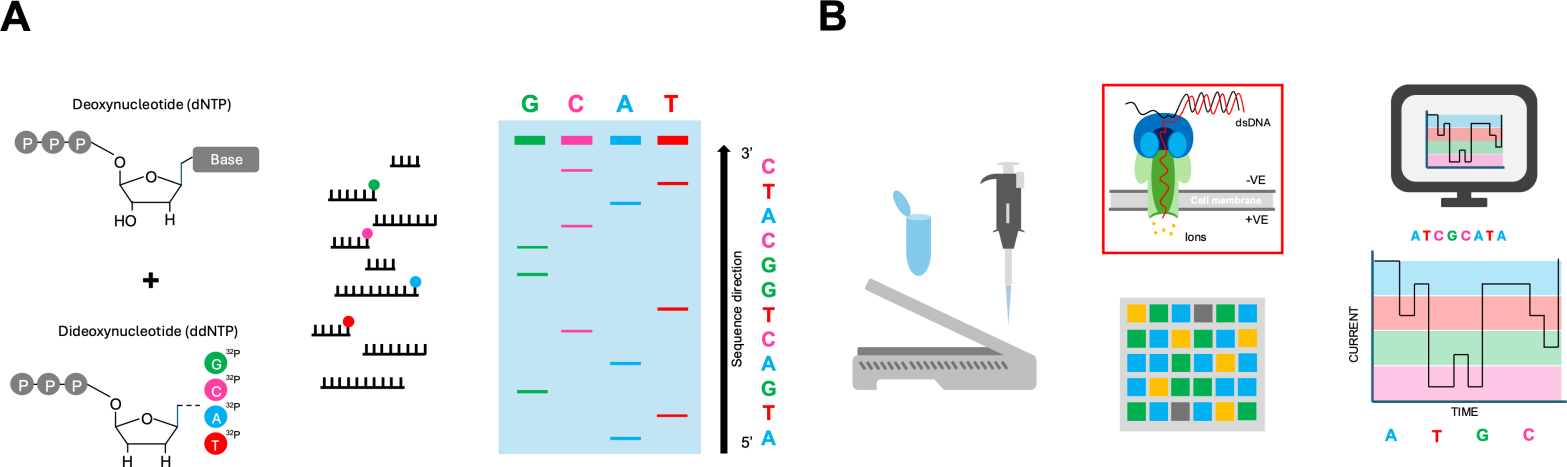
(A) Sanger sequencing using phosphorus-radioisotope-labelled chain-terminating dideoxynucleotides (ddNTPs) lacking a 3′ hydroxyl group necessary for chain polymerisation. Samples are subsequently analysed using denaturing polyacrylamide gel electrophoresis. (B) Oxford Nanopore sequencing and base-calling. Base identification is performed via the detection of changes in ionic charge and employs computational methods for current-to-base conversion.

Sequencing technologies have advanced significantly since the introduction of ‘First Generation’ DNA sequencing methods in 1977. The development of Maxam–Gilbert sequencing [18,19] by Allan M. Maxam and Walter Gilbert, and ‘Sanger Sequencing’ [20,21] by Frederick Sanger, Steve Nicklen and Alan Coulson, collectively constituted a watershed moment in life science research. Due to its greater technical simplicity and lesser reliance on hazardous reagents, Sanger sequencing subsequently became the first widely adopted DNA sequencing technology (Figure 2A). Despite this, limitations were present when compared with current sequencing technologies, including small read lengths, poor coverage and modest throughput. In combination, these factors result in low confidence sequence accuracy and challenges in the detection of both small- and large-scale sequence variation across genomes, important concepts in both molecular evolution and disease pathogenesis.

Most recently, Next-Generation Sequencing (NGS) methods, including Illumina, PacBio and Oxford Nanopore Technologies, have come to dominate the field of genome sequencing (Figure 2B). Leveraging powerful computational tools, NGS methods have revolutionised genomics with massive parallelism, scalability and speed, outperforming both Sanger and Maxam–Gilbert sequencing by an unprecedented degree [22]. NGS technologies offer the ability to sequence millions of DNA fragments per run at relatively low cost and with high accuracy. Together, these approaches afford access to read lengths from 50 to 300 (short reads) to 100,000 s of bases (ultra-long reads) [23–25]. The complementarity of sequence-by-synthesis methods such as Illumina with nanopore detection as pioneered by Oxford Nanopore Technologies (Table 1) has unlocked a raft of new applications in both research and clinical settings [26]. Due to these advances, the constant improvement and availability of sequencing technologies have resulted in the generation of an almost incomprehensible quantity of sequence data. Inevitably, the ability of downstream analytical tools to process and analyse this data has become a bottleneck, with the integration of AI-driven computational tools now considered integral in addressing this challenge. The use of AI is becoming a requirement for efficient data management and interpretation rather than a commodity in 4IR.

**Table 1:** Comparisons of First Generation and Next Generation sequencing platforms, including method, read length, coverage, throughput, accuracy, variant detection capabilities, cost and run time. SNP, single nucleotide polymorphism; indels, insertions/deletions; MEIs, mobile element insertions. 18,20,24,25.

Sequencing platform	Sanger	Maxam–Gilbert	Illumina	Oxford Nanopore
Sequencing method / Chemistry	Dideoxy chain-termination (Sanger)	Chemical base-specific cleavage sequencing	Sequence by synthesis	Single-molecule nanopore sequencing
Read length	700–1000 bp	~100 bp	22–301 bp	10 kb–4 Mb
Typical coverage	Low	Low	High	Moderate–High
Throughput	Low	Very low	Very high	Moderate–High
Accuracy	99.99%	99%	99.9%	90–98%
Variant detection	SNPs, small indels	SNPs	SNPs, small indels	SNPs, small indels, structural variants, MEIs
Cost	High	High	Low	Moderate
Run time	Days	Days	Hours	Hours

**Figure 3:**
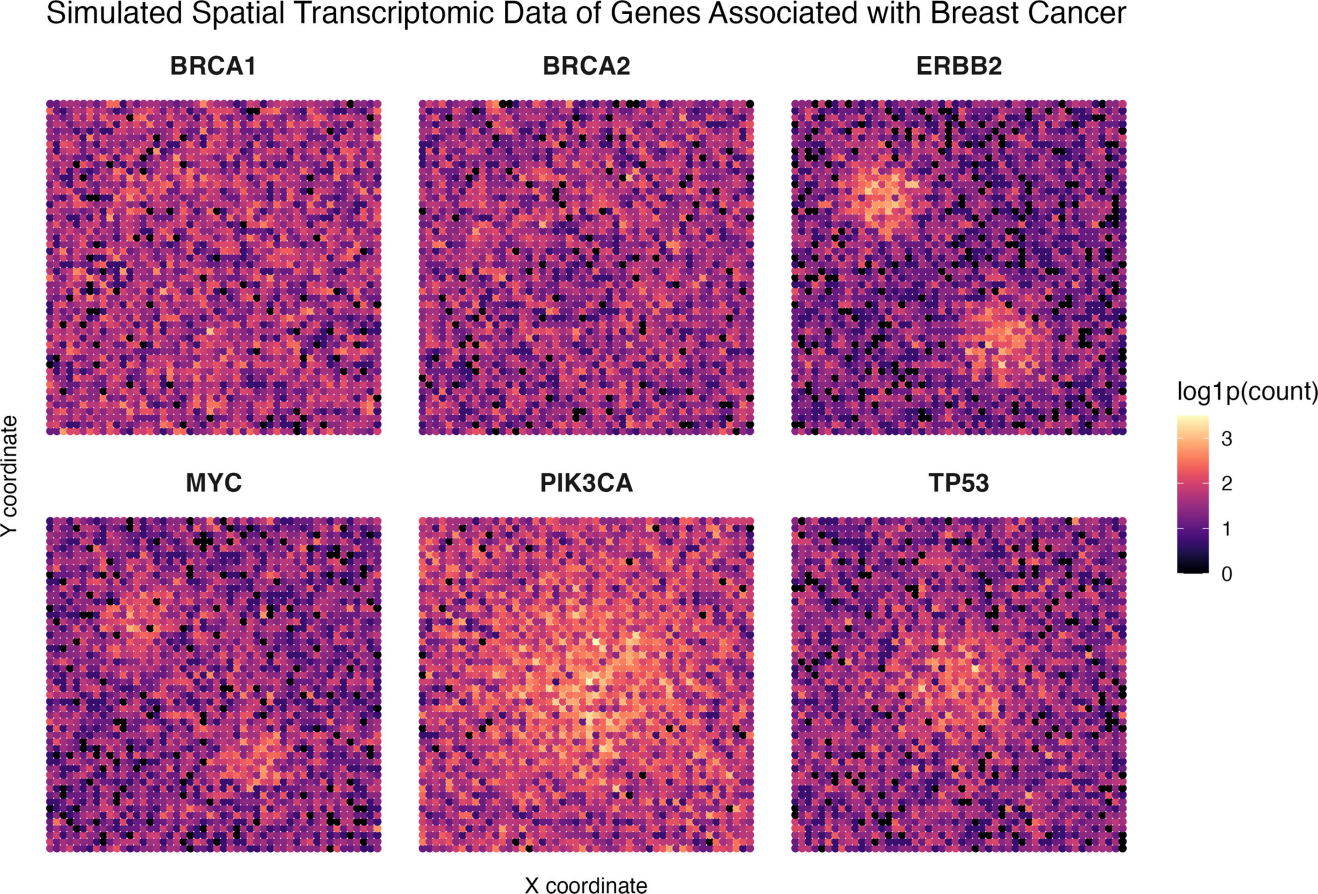
Simulated representation of spatial transcriptomics of breast cancer gene expression in the tumour region. Pseudo-data were generated in R using estimated gene expression levels of BRCA1, BRCA2, ERBB2 (HER2), MYC, PIK3CA and TP53 based on their patterns in real breast cancer cases. Each of the listed genes is associated with the development, progression and diagnosis of breast cancer and was used as close approximations for the purposes of this simulation [29–31].

Beyond sequencing, AI-enabled technological advances are accelerating progress in other areas of molecular life science research [27]. Recently, spatial transcriptomics (ST) has emerged as a powerful approach for studying localised gene expression in tissues and organs. This approach is providing new insights into biological processes within a spatial context. This context is essential for understanding disease pathogenesis and progression [28]. One such application of this technology is guiding patient treatment in cases of breast cancer, in which malignant cell location and interactions within the tumour microenvironment (TME) affect susceptibility to anticancer therapeutics. The use of ST within cancer research, particularly breast cancers as described in Figure 3, serves to highlight the importance of the spatial context of cancerous cells and their molecular interactions within the TME, specifically in informing treatment choice and administration [32]. When combined with AI, ML and DL, ST enables early disease detection and predictive modelling of pathogenesis, particularly, though not exclusively, in cancer patients [33–35]. Integration of AI within ST has yielded many positive outcomes, from increased histopathological interpretation of cancer progression to accurate predictions of patient prognosis [36].

AI models are capable of inferring tissue architecture to reveal tumour cores, invasive fronts and immune infiltration zones. These include graph-based learning methods such as graph neural networks, which leverage multi-layer biological information to generate digital descriptions of pathology and histopathology, comprising nodes relating to specific cells, tissues and genes [37]. Attached to these nodes are edges, which describe the relationships between the nodes, their spatial proximities and their molecular similarities. Additionally, latent time models which infer temporal trajectories, and by extension progression of cancers using gene expression patterns, can be used to trace the transition of healthy cells into malignant states, predicting both their spatial origin and likely timeline [38]. This integration of spatial data with AI is rapidly emerging as a research frontier, delivering important new insights and providing researchers with a greater understanding of the spatiotemporal resolution of disease pathogenesis.

Despite AI’s potential to positively affect clinical decision making, regulatory expectations relating to its use in medical contexts are yet to be comprehensively defined. As AI-guided analysis of an individual’s personal biomedical data becomes increasingly commonplace, it becomes increasingly important to introduce regulations that securely protect this information. Any data relating to an individual who may need or who is undergoing medical treatment is, by its nature, vulnerable [39,40]; thus, the implementation of formal guidance on the acceptable and ethical use of AI in relation to the interrogation of this data is of paramount importance [41].

### Multi-omics

**Figure 4:**
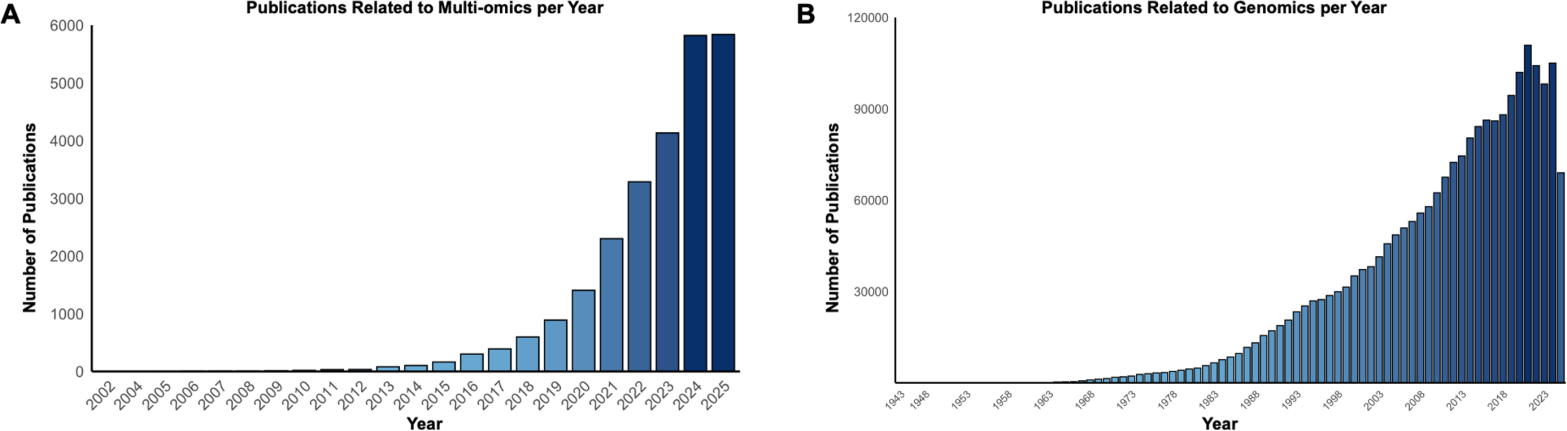
Comparison of published scientific literature as accessible via PubMed, relating to (A) ‘multi-omics’ and (B) ‘genomics’, from 2002 to 2025. The largest increase in publications related to multi-omic studies occurs between 2022 and 2025, and is overshadowed by the number of genomics publications in the same timeframe.

Omics is defined as the collective study of the entire set of biomolecules associated with a specific biological sample, and the investigation of how these molecules affect biological form and function [42]. An example of this is genomics, in which the entire genome (DNA) of an organism is studied. Multi-omics refers to the integrated analysis of biological datasets from various ‘omics’ layers, including genomics, metabolomics, transcriptomics, epigenomics and proteomics. Collated data are then used to generate high-resolution multidimensional views of cellular heterogeneity and function [43,44]. The first multi-omics study was published in 2002 and describes the relationship between the activity and structure of the genome, and the negative effects of biological agents upon it, an approach termed therein as toxicogenomics [45]. In this paper, Aradema and Macgregor described their experimental approach as an integrative analysis using data generated from other analyses, namely genomics, transcriptomics, metabolomics (previously metabonomics) and proteomics. Since its initial conception, this field has witnessed significant advances [46]. The increase in multi-omic studies and widespread use of this approach is apparent from the significant increase in scientific literature published during the last 2 years (Figure 4A), as compared, for example, with the number of published manuscripts focusing exclusively on genomics (Figure 4B).

In addition to multi-omics, single-cell sequencing has become an increasingly prominent area of AI-enabled research. This method was first reported by Tang and colleagues in 2009, who successfully introduced one of the first single-cell RNA sequence assays. In this study, successful gene expression profiling was performed on a single mouse blastomere, representing a milestone for low-input transcriptomics and providing a foundation for later developments in single-cell sequencing technology [47]. Single-cell multi-omics is a rapidly advancing field that marries single-cell RNA sequencing (scRNA-seq) with complementary omics methods, followed by a combined analysis of the resulting experimental data sets. When coupled with AI-driven analytical tools and DL/ML models, this approach enables deeper insights into complex biological processes, including disease pathogenesis, uncovering rare cell types, dynamic cell states and regulatory networks [48].

Multi-omics has evolved over the last two decades from its earliest use in describing toxicogenomics to its most recent applications in the study of complex biological systems. Integration of multiple molecular layers has provided a vastly deeper understanding of biological processes and has enabled comprehensive analyses to be performed of complex systems. Importantly, this approach provides considerably greater insights than can be achieved using any single ‘omics’ method in isolation. The addition of single-cell sequencing, namely scRNA-seq, has further increased the level of granularity to which each analysis can be performed, making it now possible to capture heterogeneity and dynamic biological states, such as those common to cancer progression. Additional AI pipelines in mass multi-omic and scRNA-seq data analysis have paved the way for future advances, as technology continues to improve at an accelerated rate with enhanced through-put, sensitivity and methods of data integration, AI and multi-omics will undoubtedly remain at the forefront of precision therapeutics and cancer diagnostic approaches, amongst others.

Whilst major advances have been made, it remains critical to recognise the limitations of AI and in particular its ability to perform decision-making tasks that require ethical reasoning, cultural understanding or subjective evaluation [49]. It is uncontested that AI can acquire and summarise large complex datasets at extraordinary speeds; however, it currently lacks the discretion necessary to undertake nuanced decision-making. Unlike humans, who are able to provide objective insights from past experiences, AI models generate results derived exclusively from the data that they have been provided with. In the context of multi-omics, this comprises layers of biological data spanning multiple ‘omic’ analyses. Missing data, annotations or deficiencies in data quality are thus not considered by AI, which is in direct contrast with human processing. This lack of discretion and experience-based decision making has to date precluded the implementation of exclusively AI-based multi-omics analysis workflows.

The high dimensionality of multi-omics data, comprising tens of thousands of individual variables, but present in relatively small sample sets, generates an additional barrier to data integration using AI. Given the requirements of AI and ML models for complete datasets [50], the accurate integration of high dimensionality data subsequently represents a significant challenge for AI analysis, the so-called Curse of Dimensionality [51]. Challenges of bias and fairness in AI processes, together with inherently high dimensionality data, can result in fundamentally discriminatory outcomes. Whilst the integration of AI models has expanded the scope of multi-omic studies, their use has highlighted the need to take into account the differences between digital and biological cognition [52].

### Lab-in-the-loop: research in the 4IR

Unquestionably, the transition to 4IR will continue to change the way scientific research is undertaken. AI and ML models are growing increasingly competent in pattern recognition, predictive abilities and data analysis [53,54]. Harnessing this technology will be paramount in driving future discoveries in the molecular life sciences. For example, Kooi et al. recently reported a state-of-the-art deep-learning AI model capable of cancer detection in mammographic exams, which was found to outperform a specialised certified radiologist [55]. This model was trained using 45,000 images from mammograms to identify signs of breast cancer and was more accurate at discriminating between cancerous and non-cancerous regions with an area under the curve of 0.9–0.941. In another example, Penadés and colleagues used a novel AI system, named AI co-scientist, to generate hypotheses relating to the mechanism by which capsid-forming phage-inducible chromosomal islands spread amongst bacterial populations [56]. Without access to the research groups’ data, the AI model produced a hypothesis which predicted their experimentally confirmed mechanism. Seemingly, AI’s capabilities have expanded to be able to creatively generate both novel and accurate hypotheses and may even suggest avenues not otherwise considered. Importantly, however, as AI models are built on training data provided by the user, should that data carry a bias, then the resulting model will subsequently present those biases. For example, one study revealed that clinicians may have overlooked positive results for African Americans as they assumed the model was less accurate for these individuals. However, their low positive rate was a direct consequence of the limited representation of these individuals in the original study upon which the AI model was based [12,57].

**Figure 5:**
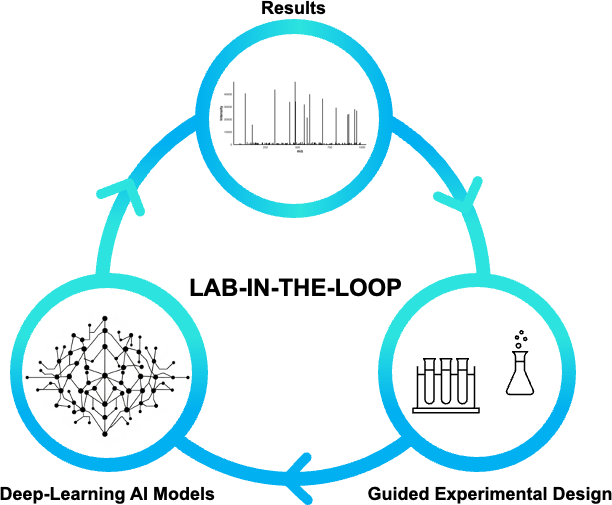
Lab-in-the-loop model. Data are used as an input for an AI to predict outcomes, formulate hypotheses, optimise protocols and suggest further experiments. The cycle continues iteratively until an optimum outcome is achieved.

It is clear that AI is set to have an increasingly significant role in molecular life science research. Consequently, there is a growing appetite to integrate these AI-based approaches into every aspect of the research process. This is perhaps typified by Genentech’s lab-in-the-loop (LitL) principle. The American biotechnology company describes LitL as a ‘closed self-feedback loop’. Data are fed to trained models, which then suggest and predict further experiments to be undertaken. The resultant data from these experiments feeds directly back into the loop, resulting in iterative cycles of hypothesis generation, experimentation and data analysis. This approach provides a closed-loop method of self-optimisation, with minimal human input (Figure 5) [58].

Currently, the LitL principle is being used predominately for the purpose of drug discovery; however, Genentech recognises that ‘a true impact will require a shift across all R&D to become part of lab-in-the-loop’ [59]. Life sciences research in 4RI appears destined to transition towards this approach, with many labs having already introduced automated systems, and in some instances, undertaken feasibility studies exploring ‘self-driving’ laboratories. Conventional laboratory research relies on human labour, with its associated susceptibility to make mistakes [60]. In contrast, automated systems can undertake repetitive laboratory tasks with minimal human intervention and in a largely error-free manner, enabling reliable high-throughput experimentation to be performed. Some laboratories have begun trailing this form of 4IR-enabled research. For example, one UK-based laboratory successfully employed an autonomous robot which displayed astonishing research productivity. Operating for over 21 hours a day, performing 100 experiments per day, the ‘automated researcher’ was estimated to be 1000 times faster than a human scientist [61,62]. It appears increasingly likely that in the not-too-distant future, life science research laboratories will transition to become predominantly automated enterprises, with the ability to formulate research questions, design experiments, and collect and analyse data in a largely iterative fashion with minimal requirement for human input. Without question, the coming decade will witness a fundamental shift in how laboratory research is undertaken in the molecular life sciences.

**Summary**

- AI technologies are performing at levels equivalent to, or exceeding, those of trained specialists.
- AI is built on potentially biased data and therefore can present the same biases.
- There is a requirement for formal regulation surrounding AI to maintain integrity and protect the confidentiality of personal data.
- Exponential improvements in sequencing technologies have expanded our ability to produce genomic information to an unprecedented extent.
- Deconvoluting this data in a meaningful way is increasingly reliant on the use of AI-based methods.
- Multi-omics has emerged as a powerful tool to integrate multiple forms of molecular-level data, deepening insights into fundamental and applied bioscience questions.
- Existing computational approaches address only parts of the problem of multi-omic data integration, and a complete solution that enables smooth integration of genomic, transcriptomic, proteomic, metabolic and spatial information does not yet exist.
- AI and automation are facilitating self-optimising laboratory experimentation to be undertaken and likely represent the future trajectory of research practice in the life sciences.

## Open Access

This article has been published open access under our Subscribe to Open programme, made possible through the support of our subscribing institutions, learn more here: https://portlandpress.com/pages/open_access_options_and_prices#conditional

## Abbreviations

AI: artificial intelligence
DL: deep learning
4IR: Fourth Industrial Revolution
LitL: lab-in-the-loop
ML: machine learning
NGS: next generation sequencing
ST: spatial transcriptomics
TME: tumour microenvironment
